# Use of a Yeast tRNase Killer Toxin to Diagnose Kti12 Motifs Required for tRNA Modification by Elongator

**DOI:** 10.3390/toxins9090272

**Published:** 2017-09-05

**Authors:** Constance Mehlgarten, Heike Prochaska, Alexander Hammermeister, Wael Abdel-Fattah, Melanie Wagner, Rościsław Krutyhołowa, Sang Eun Jun, Gyung-Tae Kim, Sebastian Glatt, Karin D. Breunig, Michael J. R. Stark, Raffael Schaffrath

**Affiliations:** 1 Institut für Biologie, Martin Luther Universität Halle-Wittenberg, Weinbergweg 10, 06120 Halle/Saale, Germany; constance.mehlgarten@genetik.uni-halle.de (C.M.); h.prochaska@nomadbioscience.de (H.P.); Melanie.Wagner@oncotec.de (M.W.); karin.breunig@genetik.uni-halle.de (K.D.B.); 2 Institut für Biologie, FG Mikrobiologie, Universität Kassel, Heirich-Plett-Str. 40, 34132 Kassel, Germany; alex_hammermeister@t-online.de (A.H.); wael@uni-kassel.de (W.A.-F.); 3 Max Planck Research Group at the Malopolska Centre of Biotechnology, Jagiellonian University, 31-007 Krakow, Poland; rostyslav.krutyholova@uj.edu.pl (R.K.); sebastian.glatt@uj.edu.pl (S.G.); 4 Department of Cell Biochemistry, Faculty of Biochemistry, Biophysics and Biotechnology, Jagiellonian University, 31-007 Krakow, Poland; 5 Department of Molecular Biotechnology, Dong-A University, Busan 604-714, Korea; junse033@hanmail.net (S.E.J.); kimgt@donga.ac.kr (G.-T.K.); 6 Centre for Gene Regulation & Expression, School of Life Sciences, University of Dundee, Dundee DD1 5EH, UK; m.j.r.stark@dundee.ac.uk

**Keywords:** zymocin, ribotoxin, tRNase, Kti12, Elongator complex, tRNA anticodon modification

## Abstract

*Saccharomyces cerevisiae* cells are killed by zymocin, a tRNase ribotoxin complex from *Kluyveromyces lactis*, which cleaves anticodons and inhibits protein synthesis. Zymocin’s action requires specific chemical modification of uridine bases in the anticodon wobble position (U34) by the Elongator complex (Elp1-Elp6). Hence, loss of anticodon modification in mutants lacking Elongator or related *KTI* (*K. lactis* Toxin Insensitive) genes protects against tRNA cleavage and confers resistance to the toxin. Here, we show that zymocin can be used as a tool to genetically analyse *KTI12*, a gene previously shown to code for an Elongator partner protein. From a *kti12* mutant pool of zymocin survivors, we identify motifs in Kti12 that are functionally directly coupled to Elongator activity. In addition, shared requirement of U34 modifications for *nonsense* and *missense* tRNA suppression (*SUP4*; *SOE1*) strongly suggests that Kti12 and Elongator cooperate to assure proper tRNA functioning. We show that the Kti12 motifs are conserved in plant ortholog DRL1/ELO4 from *Arabidopsis thaliana* and seem to be involved in binding of cofactors (e.g., nucleotides, calmodulin). Elongator interaction defects triggered by mutations in these motifs correlate with phenotypes typical for loss of U34 modification. Thus, tRNA modification by Elongator appears to require physical contact with Kti12, and our preliminary data suggest that metabolic signals may affect proper communication between them.

## 1. Introduction

Zymocin, a composite chitinase and tRNA anticodon nuclease (tRNase) ribotoxin produced from the dairy yeast *Kluyveromyces lactis*, kills other yeast species including *Saccharomyces cerevisiae* [1,2]. Although zymocin is a trimeric complex (αβγ), expression of its γ-subunit (i.e., γ-toxin tRNase) alone is lethal in *S. cerevisiae*, suggesting that the α/β-subunits facilitate zymocin docking and γ-toxin delivery [2,3]. Accordingly, screens for zymocin survivors identified mutations in *KTI* (*K. lactis* toxin insensitive) and *IKI* (insensitive to killer toxin) genes that fall into non-target (class I) and toxin-target (class II) groups based on their response to conditional γ-toxin induction [3,4,5]. Class I genes operate in the synthesis of cell wall and membrane components (chitin, sphingolipids, H^+^ pump Pma1) required for zymocin binding and γ-toxin entry, and class II loci identified a toxin–target effector role for the Elongator complex (Elp1–Elp6) [2,6]. Originally, the latter had been co-purified with elongating RNA polymerase II, hence its name [7]. However, more recently a robust body of evidence indicates that its genuine role in yeast lies with tRNA modification rather than transcription elongation. Accordingly, Elongator binds tRNAs and modifies anticodon wobble uridine (U34) bases [8,9,10,11,12,13,14]. Strikingly, the methoxy-carbonyl-methyl-thio (mcm^5^s^2^) modification at U34 in some of Elongator’s tRNA substrates (including tRNA^Glu^) allows anticodon cleavage by the γ-toxin tRNase [15]. Therefore, loss of U34 modification in Elongator mutants protects efficiently against the toxin’s attack [6,15,16].

Elevated tRNA^Glu^ levels also protect against zymocin, and this effect is suppressed by overexpression of the class II gene *KTI1*/*TRM9* [15,16]. Trm9 is the catalytic subunit of a methyl-transferase (Trm9•Trm112), which methylates the preceding cm^5^(s^2^)U34 modification generated by Elongator and also contributes to zymocin sensitivity [16,17,18]. Consistently, loss of U34 methylation in *trm9* and *trm112* mutants confers resistance to zymocin and γ-toxin tRNase [18,19]. Thus, growth inhibition of *S. cerevisiae* by zymocin apparently involves restriction of U34-modified tRNAs affecting protein biosynthesis. In support of this strategy, which is shared by other microbial anticodon nucleases [20,21], zymocin depletes tRNA^Glu^ in vivo, and overexpression of tRNA ligase suppresses the tRNA attack showing that tRNA repair is able to ‘heal’ the damage in the anticodon loop [22]. Use of the γ-toxin tRNase as a tool diagnostic for Elongator activity identified additional class II gene products with related roles in tRNA modification [2,5,19,23,24,25]. Among them, a casein kinase I isozyme (Hrr25/Kti14), a protein phosphatase (Sit4) and an Elongator partner protein (Kti12) were found to constitute a complicated network that regulates the phosphorylation status of Elongator’s largest subunit (Elp1) [26,27,28]. This suggested that phosphorylation affects Elongator activity, a notion supported by data showing that Hrr25 phosphorylation sites on Elp1 are critical for U34 modification [29] and that Hrr25 association with Elongator requires Kti12 [28].

Sequence alignments between Kti12 and its plant ortholog DRL1/ELO4 suggested that both share a P-loop motif characteristic for nucleoside triphosphate (NTP) binders [30] and a putative calmodulin (CaM) binding domain (CBD) [31]. With ELO4 known to bind CaM in vitro and associate with plant Elongator in vivo [32,33], the presence of cofactor binding motifs points towards conserved roles for Kti12 and ELO4. To further analyze Kti12 requirements for Elongator activity, we studied a pool of zymocin resistant *kti12* mutants previously used [5] to identify the *KTI12* locus in yeast. We map mutations in the CBD and P-loop motifs and other regions conserved between Kti12 and ELO4 that, based on genetic and biochemical assays, trigger traits typical of Elongator-linked loss of U34 modification and defects in Elongator interaction. In summary, our data reinforce the view that Kti12 relates in function to the yeast Elongator complex and stimulates its tRNA modification activity.

## 2. Results and Discussion

Based on its similarity to yeast Kti12, the *Arabidopsis* protein ELO4 was previously proposed to play an Elongator-related role in plants [24,32]. This was confirmed when ELO4 was shown to associate with TAP-tagged plant Elongator and *ELO4* mutations found to cause phenotypes typical of plant Elongator mutants [33]. In addition, alignments between Kti12 and ELO4 proteins from fungal and plant species (Figure S1) revealed several regions of high similarity. Among these are P-loop motifs, putative calmodulin (CaM) binding domains (CBDs) [24,31,32] and other homology regions (Figure S1). While P-loops may be indicative of NTP binding [30], the CBDs likely mediate the demonstrated in vitro binding of ELO4 to CaM [31,32]. In comparison to strong CBDs (type 1-5-10) used, e.g., in tandem affinity purification (TAP) protocols [34], the CBDs in Kti12 or ELO4 (type 1-8-14) show weaker CaM affinity [35]. In summary, similarity in sequence and domain organisation may indicate that the motifs found in Kti12 and ELO4 are evolutionarily conserved because they functionally interact with Elongator.

### 2.1. A kti12 Mutant Pool Reveals Conserved Kti12 Motifs with Elongator-Related Roles

**Figure 1:**
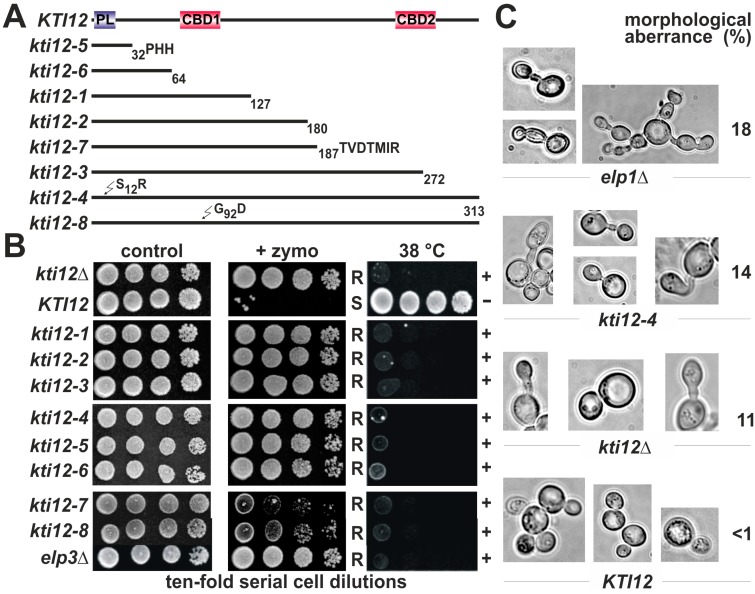
A set of *kti12* mutants uncovers a phenotypic signature typical of Elongator defects. (**A**) compilation of *kti12* mutants including mutation mapping and alignment to Kti12 domain organization (for details, see Figure S1); (**B**,**C**) phenotypes shared between *kti12* and Elongator mutants. Growth assays (**B**) with yeast strains of the indicated backgrounds at 30 °C (control), elevated temperature (38 °C) or in the presence of 45% [*v*/*v*] zymocin (+zymo). Traits typical of Elongator mutant (*elp3*∆) include zymocin resistance (R) and thermosensitivity (+); zymocin sensitivity (S) and thermotolerance (−) denote wild-type (*KTI12*) phenotypes; (**C**) phase contrast microscopy to monitor cell/bud morphology in the indicated strain backgrounds.

Given that *KTI12* deletions cause Elongator defects [6,24,36], we analysed a previously isolated pool of zymocin resistant *kti12* mutants [5] in greater detail (Figure 1A). Single amino acid substitutions within full-length Kti12 were mapped to the P-loop (*kti12-4*: S12R) and CBD1 (*kti12-8*: G92D) motifs (Figure 1A). In addition, *nonsense* (*kti12-1*, *kti12-2*, *kti12-3* and *kti12-6*) or frame-shift (*kti12-5* and *kti12-7*) mutations led to truncations resulting in deletion of various invariant Kti12 regions including one or both CBD motifs (Figure 1A). Following assays diagnostic for Elongator-minus phenotypes, all *kti12* mutants were found to be zymocin resistant, hypersensitive to 7.5 mM caffeine (data not shown) and sensitive to temperatures above 38 °C (Figure 1B). Collectively, these traits phenocopy *kti12*Δ and *elp3*Δ strains (Figure 1B), reinforcing the view that Kti12 and Elongator are closely related in function [6,24,36].

In addition, significant proportions of *kti12*∆ (11%), *kti12-4* (14%) and *elp1*∆ (14%) mutants showed aberrantly elongated cells/buds (Figure 1C), morphological defects previously observed in association with Elongator or related tRNA modification mutants including *kti14/hrr25* cells [25,28,37]. Consistent with Elongator mutations known to suppress thermosensitive growth of *sec2-59*^ts^ and *sec12-4*^ts^ mutants at 31 °C and 33 °C, respectively [38,39], *sec* mutant rescue was also seen with the *kti12-4* and *kti12-8* alleles (Figure S2), although to a lesser extent than *elp1*∆. In summary, an Elongator-like phenotypic signature of *KTI12* loss-of*-*function alleles documents the usefulness of zymocin as a tool diagnostic for Kti12 protein motifs that are important for Elongator activity.

### 2.2. Conserved Kti12 Motifs Support Elongator-Dependent tRNA Suppression

**Figure 2:**
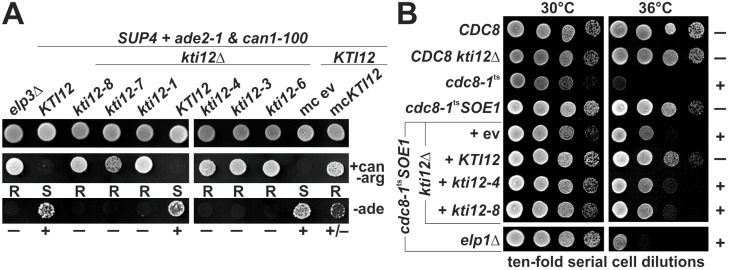
*KTI12* gene mutations and copy number interfere with U34 tRNA suppressors. (**A**) tRNA *nonsense* suppression. In the indicated genetic backgrounds, *can1-100* and *ade2-1* *ochre* read-through by *SUP4* was phenotypically assessed by canavanine sensitivity/resistance (S/R: middle panel) on canavanine supplemented medium lacking arginine (+can −arg) or by adenine proto-/auxotrophy (+/−: bottom panel) on minimal medium without adenine (−ade). Growth control (top panel) involved yeast peptone dextrose (YPD) rich medium. Empty vector (ev) served as reference for multi-copy (mc) *KTI12*; (**B**) *SOE1* tRNA *missense* suppression. The indicated strains were grown at temperatures permissive (30 °C) or restrictive (36 °C) for *cdc8-1*^ts^ cells. Note that *ELP1* and *KTI12* gene mutations affect (+) rescue of *cdc8-1*^ts^ cell growth at 36 °C (−) by the tRNA suppressor *SOE1*.

Physiologically, Elongator-dependent tRNA modification fine-tunes tRNA decoding [40,41,42,43] and maintains ribosomal reading frame accuracy during translation [44,45,46,47]. In support, stop codon read-through by U34 carrying tRNA suppressors (*SUP4*; *sup9e*) requires Elongator function [8,16,39]. Inspired by previous data showing that some class II *kti* mutants also have tRNA modification defects [8,19], we compared *SUP4* read-through between members of the *kti12* mutant collection and an *elp3*Δ knock-out in reporter strain UMY2893. The latter maintains *ochre* (UAA) alleles of *ADE2* (*ade2-1*) and *CAN1* (*can1-100*), which upon suppression by *SUP4*, confer adenine prototrophy (Ade^+^) and canavanine sensitivity (Can^S^), respectively [8,16]. In agreement with previous reports, *SUP4* read-through of *ade2-1* and *can1-100* was abolished by *ELP3* gene deletion yielding adenine auxotrophic (Ade^−^) and canavanine resistant (Can^R^) *elp3*∆ cells (Figure 2A). Notably, both traits (Ade^−^, Can^R^) were copied by all the *kti12* mutant alleles (*kti12-1*, *kti12-3*, *kti12-4*, *kti12-6*, *kti12-7* and *kti12-8*) we tested (Figure 2A) and complemented after reintroducing a *KTI12* wild-type gene into *kti12*∆ cells (Figure 2A). The latter finding supports our notion that recessive *KTI12* loss-of*-*function alleles trigger Elongator-linked tRNA modification defects and phenotypes in yeast (Figure 1 and Figure 2).

Consistent with our previous observation that Elongator dependent processes (including e.g., zymocicity) can be suppressed by overexpression of *KTI12* [5,26], multi-copy *KTI12* also countered tRNA suppression of *can1-100* by *SUP4* (Figure 2A). As for *ade2-1*, however, multi-copy *KTI12,* allowed weak growth in medium lacking adenine, a trait in between the phenotypes of wild-type (Ade^+^) or *elp3/kti12* mutant (Ade^−^) cells (Figure 2A). The latter read-out may indicate that the two *ochre* reporters differ in their level of sensitivity level to Elongator defects. Presumably, a weaker Ade^+^ trait could be interpreted in a way that increased intracellular Kti12 levels also decrease the tRNA modification activity of Elongator (Figure 2A). It will be interesting to address this scenario in greater detail in future studies using quantitative *lacZ*^ochre^ read-through assay or direct LC-MS/MS measurements [29,48,49]. These techniques will allow comparison of the relative abundance of Elongator dependent U34 modification levels between mutants lacking Kti12 and cells expressing Kti12 at elevated levels from either single-copy or multi-copy plasmids.

In addition, we observed that similar to *kti12*∆ and *elp1*∆ cells, *kti12-4* and *kti12-8* alleles also antagonized tRNA *missense* suppression of *cdc8-1*^ts^ by *SOE1* (Figure 2B), a mutant tRNA^Glu^ that reads Lys codons in a fashion dependent on U34 modification by Elongator [39,50]. Hence, proper Elongator and Kti12 activity are required for *SOE1* to rectify Glu to Lys substitutions in *cdc8-1*^ts^ cells and thus suppress their thermosensitivity at 36 °C (Figure 2B). Together with previous data showing that *missense* (*SOE1*) and *nonsense* (*SUP4*; *sup9e*) suppressor tRNAs depend on Elongator [8,16,39], Kti12 can thus be considered to promote suppressor tRNA functionality, most likely through its regulatory role in Elongator’s tRNA modification reaction.

### 2.3. Kti12 Expression and Interaction Profiles

**Figure 3:**
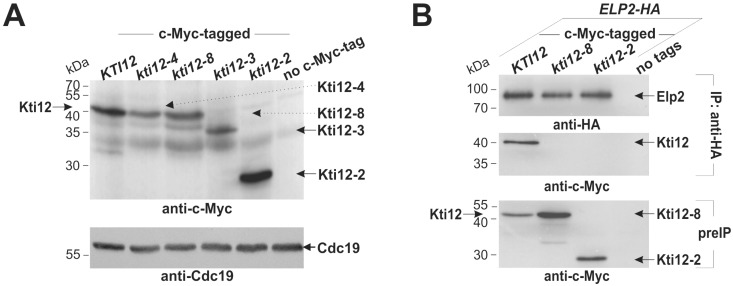
Kti12 expression and Elongator interaction studies. (**A**) expression of Kti12 and its mutated variants. The indicated *KTI12/kti12* alleles were genomically c-Myc-tagged and each gene product identified (top panel) in anti-c-Myc Western blots of total yeast protein extracts. An independent, anti-Cdc19 blot served as internal control (bottom panel); (**B**) elongator interaction studies with Kti12 and its mutants. Strains co-expressing Elp2-HA and c-Myc-tagged Kti12, Kti12-2 or Kti12-8 were subjected to anti-HA immune precipitation (IP). The IPs were probed for Elp2-HA (top panel) or Kti12-c-Myc (middle panel) with anti-HA or anti-c-Myc antibodies, respectively. Total protein extracts (preIP, bottom panel) probed with anti c-Myc antibodies served as Kti12 input control.

To examine whether and how the mutated Kti12 proteins are expressed, we subjected *kti12-2*, *kti12-3*, *kti12-4* and *kti12-8* alleles to genomic c-Myc-tagging. Following Western blot analyses, all epitope-tagged Kti12 variants were found to be produced at the expected molecular weights (Figure 3A); Kti12-4 and Kti12-8 are full-length (~40 kDa) while Kti12-2 (~26 kDa) and Kti12-3 (~35 kDa) represent C-terminal truncations (Figure 3A). In addition, while Kti12-3 and Kti12-4 levels were reduced, Kti12-2 and Kti12-8 expression compared favourably with Kti12 wild-type levels (Figure 3A). To avoid abundance related secondary effects, we conducted subsequent analyses with the latter two mutants. Since Kti12 and Elongator associate with each other [6,24,28,36], we examined whether inappropriate interaction between both may underlie the loss-of-function traits observed in *kti12-2* and *kti12-8* cells (Figure 1 and Figure 2). Therefore, c-Myc-tagged *KTI12, kti12-2* and *kti12-8* alleles (Figure 3A) were each co-expressed with an HA-tagged copy of the *ELP2* gene for co-immune precipitation (IP) assays (Figure 3B). Unlike the strong interaction observed between wild-type Kti12 and Elp2 (Figure 3B), the Elongator subunit failed to co-precipitate Kti12-2 and Kti12-8 (Figure 3B) and both mutants hardly co-purified Elp2 in reciprocal IPs (data not shown). This indicates that a proper interaction with Elongator requires both the structural integrity of CBD2 and conserved Kti12 residues further downstream (truncated in *kti12-2* cells) and an intact CBD1 motif (mutated in *kti12-8* cells) (Figure 1A). Whether the G92D mutation (Figure 1A) affects Elongator interaction in *kti12-8* cells through changes in local hydrophobicity (Figure S4) is an option currently under investigation. In summary, loss of Elongator binding due to mutations in specific and putative functional Kti12 domains correlates with Elongator-minus traits (Figure 1 and Figure 2). These observations again support our previously presented working hypothesis that direct physical contact with Kti12 is required for Elongator’s ability to modify tRNA anticodons [6,28,29].

### 2.4. Affinity of Kti12 for CaM

**Figure 4:**
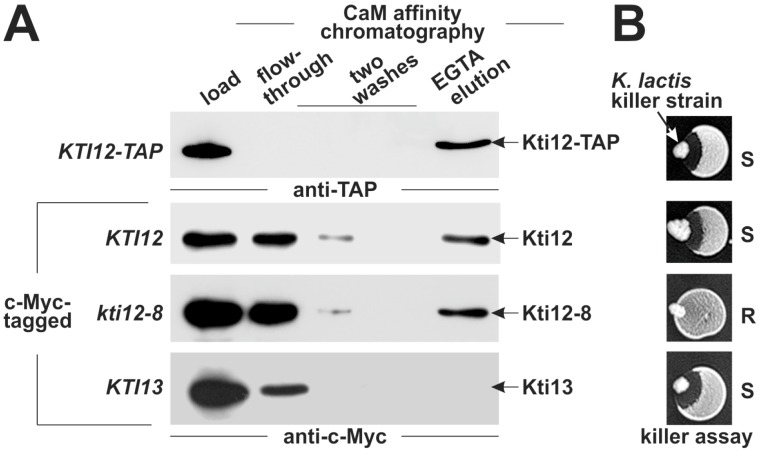
CaM binding by Kti12 in vitro. (**A**) CaM affinity chromatography of Kti12 and Kti13 variants from the indicated genetic backgrounds. Equal proportions of input (load), unbound (flow-through; washes) and eluted (EGTA elution) protein fractions were subjected to anti-TAP (top panel) and anti c-Myc Western blots (other panels); (**B**) killer eclipse assays between the same *S. cerevisiae* strains used for CaM affinity chromatography and a *K. lactis* zymocin producer (killer strain). ‘S’ or ‘R’ denote zymocin sensitivity or resistance.

As the putative CBDs may be involved in Kti12 function (Figure 2 and Figure 3) [24,31,32], we examined next whether yeast Kti12 binds to CaM. In detail, we subjected total yeast extracts containing c-Myc-tagged Kti12 or Kti12-8 to CaM Sepharose affinity chromatography. Extracts containing Kti13-c-Myc, a protein without any CBD motif, or Kti12-TAP with a high-affinity CaM-tag were used as negative and positive controls, respectively (Figure 4). Protein bound to the CaM matrix was eluted with EGTA to chelate calcium and analyzed in Western blots. Both wild-type Kti12-c-Myc and Kti12-8-c-Myc could be detected in the eluate, whereas the negative control Kti13-c-Myc could not (Figure 4). The flow-through fractions indicate that, as expected, the affinity of Kti12-c-Myc is lower than that of Kti12-TAP, which carries an additional CBD derived from the TAP tag [34]. Whether the second CBD (Figure S1) might contribute to residual binding of the *kti12-8* mutant to CaM is not known. Collectively, our data indicate that Kti12 and Kti12-8 bind CaM in a calcium sensitive manner. Although they cannot unambiguously provide evidence that the Gly-92 residue in Kti12 is part of a bona fide CBD, they show that Kti12 has CaM affinity, a finding consistent with demonstrated in vitro binding of plant ELO4 to CaM [32]. Whether the ability of Kti12 to bind CaM contributes to Elongator function has to await further investigation.

### 2.5. Cross-Complementation Analysis between KTI12 and ELO4

**Figure 5:**
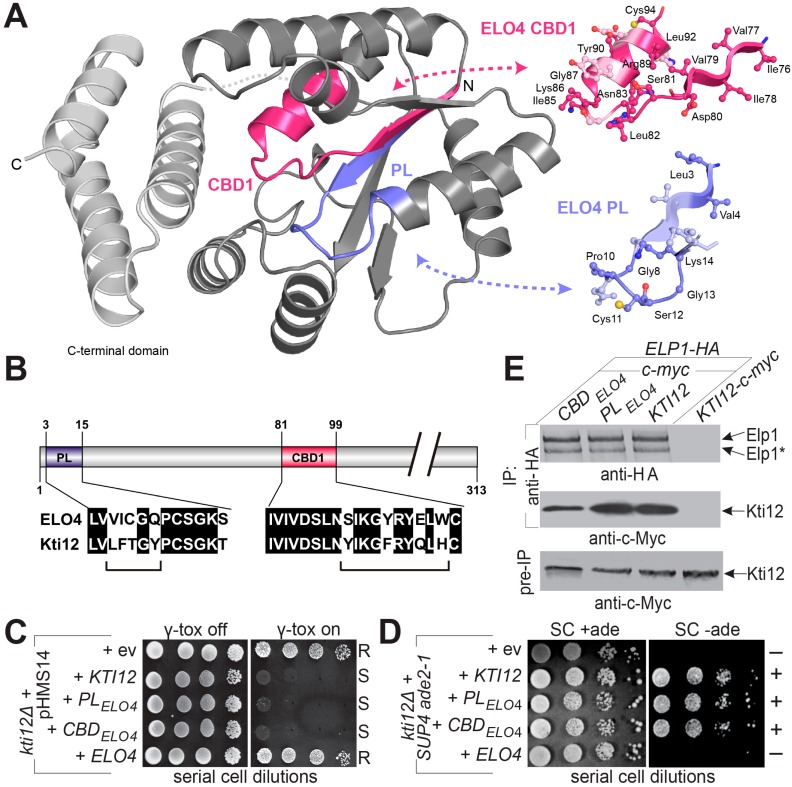
*KTI12* and *ELO4* gene shuffle analysis. (**A**) A PSTK homolog-based structural model of yeast Kti12 with highlighted conserved P-loop (blue) and CBD1 (pink) in cartoon representation. PSTK based structures of P-loop (PL) and CBD1 from ELO4 provide a structural insight into the motif swap experiment. Highly conserved residues are shown in ball and stick representation, and labelled respectively; (**B**) Indication of PL and CBD motif shuffling from ELO4 to Kti12; (**C**,**D**) cross-complementation was studied by (**C**) conditional γ-toxin tRNase expression [6] on glucose (γ-toxin: off) vs. galactose (γ-tox: on) and (**D**) *SUP4 nonsense* suppression of *ade2-1* (see also Figure 2A). R/S: γ-toxin resistance/sensitivity; +/−: adenine proto-/auxotrophy; (**E**) elongator interaction. Strains co-expressing Elp1-HA and indicated c-Myc-tagged Kti12 variants were subjected to anti-HA immune precipitation (IP). The IPs were probed with anti-HA and anti-c-Myc antibodies to check for content of Elp1-HA (top panel) and co-precipitated Kti12 material (middle panel). Total extracts (bottom panel) were probed with anti-c-Myc antibodies and served as input (preIP) control.

Based on several lines of evidence including sequence similarities (27.9% identity; 45.3% similarity, Figure S1), elongator-related functions in vivo and CaM binding in vitro (Figure 4) [31,32,33], Kti12 and ELO4 may be functional counterparts. To study structure and functional conservation between subdomains (Figure 5A), we engineered hybrid alleles of *KTI12* encoding motif-swapped proteins in which the P-loop or CBD1 motif from Kti12 is replaced with the respective ELO4 sequence (Figure 5B). After introducing these into a *kti12*∆ reporter strain, both hybrids were able to complement the loss-of function phenotype of the mutant; sensitivity to growth inhibition by the γ-toxin tRNase subunit of zymocin (Figure 5C) and *ochre* codon read-through of *ade2-1* by *SUP4* (Figure 5D) were restored. In contrast, the plant *ELO4* gene leaves γ-toxin resistance and adenine auxotrophy (Ade^−^) of *kti12*∆ cells unchanged (Figure 5C,D). Hence, the *Arabidopsis* homologue of Kti12 is unable to replace Kti12 function in yeast, whereas the hybrid proteins are functional. We conclude that sequence differences in the highly conserved motifs are unlikely to be responsible for the specificity of Kti12. Notably, the *KTI12* gene from *K. lactis*, which is more closely related to that of *S. cerevisiae,* is also unable to complement the *kti12*∆ mutation (data not shown). To address the question of whether Elongator interactions are retained in the motif-swapped Kti12-c-Myc variants, they were co-expressed with Elp1-HA, and IPs were performed with anti-HA antibodies. Elp1-HA co-precipitated native Kti12 and both hybrids (Figure 5E). Based on protein expression in the extracts prior to IP that were compared to wild-type Kti12 levels, the CBD1-swapped hybrid may be slightly reduced in Elp1 interaction. In summary, we conclude that while alleles coding for hybrids, in which conserved cofactor binding motifs of ELO4 (P-loop, CBD1) have been engineered into yeast Kti12, are functionally exchangeable, full-length plant ELO4 is not. Cross-complementation was also absent with plant *ELO4* homologs from rice (Os*ELO4*) and moss (Pp*ELO4*) (Figure S3). The incompatibility of ELO4 with Elongator function in *S. cerevisiae* contrasts with previous findings showing that *elp1*∆ mutants can be partially rescued by *ELO2*, the plant homolog of yeast *ELP1* [51]. Strikingly, the most highly conserved Elongator subunit gene (*ELP3*) gene cannot be replaced in yeast by the *Arabidopsis ELP3* homolog even though the plant subunit assembled into holo-Elongator complexes [9]. However, functional complementation in yeast was achieved when the two plant Elongator genes (*ELO2*, *ELP3*) were co-expressed in the genetic background of an *elp1*∆ *elp3*∆ double mutant [9]. Hence, non-conserved plant-specific sequences in ELO4 may mediate a selective physical or functional interaction with individual Elongator subunits, Hrr24 or Sit4 to explain the failure of cross-complementation in our gene shuffle experiments (Figure 5C,D). Regions of species-specific sequence variation may thus provide first insights into the evolutionary differences between plant ELO4 and yeast Kti12.

## 3. Conclusions

To conclude, the zymocin tRNase ribotoxin can be exploited as a molecular diagnostic tool to assign function to motifs conserved in two Elongator partner proteins, Kti12 and plant ELO4. These motifs are potentially involved in cofactor binding (nucleotide, calmodulin) and when mutated, abolish Elongator interaction and trigger phenotypes typical of tRNA modification defects in known Elongator mutants. Thus, tRNA modification by Elongator appears to require physical contact and proper communication with Kti12, a notion further supported by our findings that Kti12 and Elongator cooperate in proper performance of *nonsense* and *missense* tRNA suppressors (*SUP4*, *SOE1*). Despite their structural and functional similarities, Kti12 and ELO4 cannot be functionally exchanged between yeast and plant cells. This suggests the occurrence of species-specific barriers or evolutionary diversification of Elongator and its associated regulatory proteins between lower and higher eukaryotes. In summary, we provide further evidence for a dynamic and complicated network underlying potential regulation of the U34 modification pathway. It constitutes regulatory proteins including Kti11/Dph3, Kti12, Kti13/Ats1 and Kti14/Hrr25 [28,29,36,39,52,53,54,55] whose associations with the Elongator complex appear to be sensitive to certain metabolic signals. In support of this notion are recent findings showing that loss of U34 modifications cause altered metabolic profiles in Elongator mutants [56].

## 4. Materials and Methods

### 4.1. Yeast Strains, Media and General Methods and Plasmid Constructions

Growth of yeast strains (Table S1) was in routine YPD or SC media [57] for 3 days at 30 °C. Thermosensitivity was assessed by cultivation at elevated (31 °C, 33 °C, 36 °C, 39 °C) temperatures. Caffeine sensitivity involved YPD supplementation with 7.5 mM of the chemical (Sigma Aldrich, Taufkirchen, Germany). Table S2 lists primers used for PCR-based protocols to epitope tag and delete genes and to map *kti12* mutations [3,5] by Sanger DNA sequencing. Transformation of yeast cells with PCR products and plasmids (Table S3) was done as described [58]. Monitoring zymocin sensitivity/resistance used direct colony-colony eclipse assays [4] between *K. lactis* killer strain AWJ137 (Table S1) and *S. cerevisiae* tester strains or YPD plate assays containing 40–50% (*v*/*v*) partially purified zymocin [26]. Responses towards intracellular expression of zymocin’s lethal tRNase subunit (γ-toxin) used *GAL1*-γ-toxin expression vector pHMS14 as described [6]. *ade2-1* and *can1-100* *ochre* read-through by *SUP4* and *missense* suppression of *cdc8-1*^ts^ by *SOE1* followed previous protocols [8,16,50] as did *sec2-59*^ts^ and *sec12-4*^ts^ suppression assays [38,39]. Construction details of *KTI12* hybrid alleles, in which the P-loop and CBD domains were replaced with *ELO4* motifs (PL*_ELO4_* & CBD*_ELO4_*; Figure 5B) by two-step fusion PCR, template plasmid pDJ75 and indicated primers (Table S2), can be made available on request. Expression of *ELO4/DRL1* cDNAs from *Arabidopsis* (At*ELO4*), rice (Pp*ELO4*) and moss (Pp*ELO4*) under control of the *TDH3* promoter utilized single-copy vector pTU1 [31]. For functional analysis, *kti12* mutant and *KTI12* wild-type alleles were moved following rescue (see below) from the multi-copy backbone of pJHW27 into single-copy vector YCplac33 [59].

### 4.2. Functional Analysis of the kti12 Mutant Pool and Genomic Manipulations at the ELP and KTI Loci

To identify individual *KTI12* mutations from the *kti12* pool (*kti12-1* to *kti12-8*) (Table S1), ARB(K) member strains [3,5] raised in LL20 and KY117 backgrounds (Table S1) were subjected to gap repair in vivo using plasmids pJHW27 or pDJ40 (Table S3) restricted with *Nde*I and *Nco*I [5] or to direct *kti12* locus amplification. The latter used genomic DNA for PCR with *Pfu* polymerase (Fermentas) and primers *KTI12-P* and *KTI12-4* (Table S2). Yeast plasmids rescued in *E. coli* or generated by sub-cloning the above PCR products in TOPO pCR2.1 (Invitrogen, Thermo-Fisher Scientific, Waltham, MA, USA) were sequenced in reactions containing 4 μL of ready reaction premix (2.5×), 2 μL of BigDye terminator v1.1 sequencing buffer (5×) (Applied Biosystems, Waltham, MA, USA), 200–500 ng of plasmid DNA and 3.2 pmol of sequencing primers *KTI12-P* and *KTI12-1* to *KTI12-4* (Table S2) in a final volume of 20 μL. Following 30 cycles, the PCR reactions were subjected to ethanol precipitations and run on an ABI-Prism 377 DNA sequencer (PerkinElmer, Hamburg, Germany). Tagging of *KTI12* alleles and Elongator subunit genes (*ELP1*, *ELP2*) with c-Myc or HA epitopes essentially followed previous PCR protocols [6,24] using the S2/S3 primer pairs (Table S2) compatible with the pYM template plasmids (Table S3). *KTI12-TAP* involved the PCR protocol of Puig et al. [34] with template plasmid pBS1479 (Table S3) and up/down-*KTI12*primers (Table S2)*.* c-Myc tagging of *KTI13* was previously described [23]. *KTI12*, *ELP1* and *ELP2* gene deletions were created using published [6,24] PCR protocols, primers and marker plasmids (Tables S2 and S3). Gene deletions were physically verified by PCR on genomic DNA preparations using gene-specific diagnostic primers (Table S2) and phenotypically confirmed by zymocin assays (see above).

### 4.3. KTI12 Expression Analyses and Elongator or CaM Interaction Studies 

Immune detection of epitope-tagged Kti12 and Elongator subunits and immune precipitations (IPs) from total yeast extracts used anti-c-Myc (9E10) and anti-HA (3F10) antibodies (Roche) as previously described [6,24]. Protein loading was checked with anti-Cdc19 serum (a kind donation by Dr J. Thorner, University of California, Berkley, CA, USA), which recognizes pyruvate kinase. CaM binding assays in vitro involved TAP-tagged or c-Myc-marked Kti12 and Kti13 proteins from total yeast extracts (~2 mg) adjusted to 1 mM CaCl_2_. The material (2 mL) was mixed with CaM-Sepharose for 30 min and equilibrated with 2 mL CaM binding buffer (CBB) as described [32]. The slurry was packed onto a column, the buffer drained, and the column rinsed twice with 2.5 volumes of CBB. Elution of bound proteins was in EGTA buffer (4 mM Tris-HCl, pH 7.5, 200 mM NaCl, 1 mM MgCl_2_, 2 mM EGTA, and 0.1 mM DTT). Equal proportions of load, flow-through, wash, and elution fractions were separated by 15% SDS-PAGE, and Kti12 material was detected using anti-TAP and anti-c-Myc Western blots.

### 4.4. Kti12 Secondary Structure Predictions and Alignments with ELO4/DRL1

Secondary structure predictions for Kti12 and ELO4 proteins were obtained using the semi-automated homolog-based protein fold recognition server Phyre2.0 [60]. Best matching records, in both cases based on the crystal structure of archaeal *O*-phosphoseryl-tRNA(Sec) kinase (PSTK, PDB: 3A4M) [61], were further used to visualize Kti12 domain architecture and illustrate a motif-swap experiment (Figure 5B). All structures were rendered using PyMOL Molecular Graphics System (http://www.pymol.org/) (Version 1.8 Schrödinger, LLC, New York, NY, USA). Sequences of Kti12 or its homologs from indicated organisms were obtained from NCBI. Multiple alignments were generated using MUSCLE available at EMBL-EBI web page (http://www.ebi.ac.uk/Tools/msa/muscle/). MSA files were handled using JalView software [62]. Homolog-based model and multiple alignment were used for sequence conservation illustration with the CONSURF server (http://consurf.tau.ac.il/) [63].

## Supplementary Materials

The following are available online at www.mdpi.com/2072-6651/9/9/272/s1, Figure S1: Multiple Kti12 sequence alignment; Figure S2: *ELP1* and *KTI12* mutations rescue thermosensitivity of *sec2-59*^ts^ and *sec12-4*^ts^ mutants; Figure S3: Analysis of yeast *kti12* cross-complementation by *ELO4* plant homologs; Figure S4: Hydropathy plots of Val-82 to Lys-102 spanning regions in Kti12 and Kti12-8; Table S1: Yeast strains; Table S2: Primers; Table S3: Plasmids.
