# Supplementary material for: Use of a Yeast tRNase Killer Toxin to Diagnose Kti12 Motifs Required for tRNA Modification by Elongator
Source: Toxins (Basel). 2017 Sep 5;9(9):272. doi: 10.3390/toxins9090272 (PMC5618205; doi:10.3390/toxins9090272)
Supplement: Supplementary file 1 [file toxins-09-00272-s001.pdf]

Supplementary Materials: Use of a Yeast tRNase Killer Toxin to Diagnose Kti12 Motifs Required for tRNA Modification by Elongator

Constance Mehlgarten, Heike Prochaska, Alexander Hammermeister, Wael Abdel-Fattah, Melanie Wagner, Rościsław Krutyhołow, Sang Eun Jun, Gyung-Tae Kim, Sebastian Glatt, Karin, D. Breunig, Michael J.R. Stark and Raffael Schaffrath

1. Supplementary Tables

Table S1. Yeast strains.

Strain	Genotype	Source/Reference
Kluyveromyces lactis:		
AWJ137	α *leu2 trp1* [k1/k2] zymocin producer, killer yeast	K.D. Breunig
Saccharomyces cerevisiae:		
LL20	*MATα leu2-3, 112 his3-11, 15 can1*	M.J.R. Stark
ARB18; 46; 47; 72; 76; 78	LL20, *kti12-1; kti12-2; kti12-3; kti12-4; kti12-5; kti12-6*	[1]
KY117	*MAT***a** *ura3-52 trp1-Δ1 lys2-801 ade2-101 his3Δ200*	M.J.R. Stark
ARBK53; 67	KY117, *kti12-7; kti12-8*	[1]
SSY1	LL20, *KTI12-c-myc::SpHIS3*	This study
SSY2	ARB46, *kti12-2-c-myc::SpHIS3*	This study
SSY3	ARB72, *kti12-4-c-myc::SpHIS3*	This study
SSY4	ARBK67, *kti12-8-c-myc::SpHIS3*	This study
SSY5	ARB47, *kti12-3-c-myc::SpHIS3*	This study
W303-1A	*MAT***a** *ade2-1 his3-11, 15 leu2-3, -112 trp1-1 ura3-1 can1-100*	Lab stock
CY4209	W303-1A, *SSD1-v1*	Lab stock
SSY8	CY4209, *ELP2-HA::KlTRP1 KTI12-c-myc::SpHIS3*	This study
SSY16	CY4209, *ELP2-HA::KlTRP1 kti12-2-c-myc::SpHIS3*	This study
SSY12	CY4209, *ELP2-HA::KlTRP1 kti12-8-c-myc::SpHIS3*	This study
RZY06	CY4209, *KTI13-c-myc::SpHIS3*	R. Zabel
TOT4TAP	CY4209, *KTI121-TAP::KlTRP1*	L. Fichtner
DJY104	CY4209, *kti12Δ::KlLEU2 ELP1-HA::KlTRP1*	[2]
W303-1B	W303-1A, *MATα*	Lab stock
UMY2893	W303-1B, *SUP4*	[3]
UMY2916	UMY2893, *elp3Δ::kanMX4*	[3]
UMY2938	UMY2893, *kti12Δ::kanMX4*	[2]
126	*MATα trp1–289 ura3–52 leu2–3/112 can1 ade1,2 CDC8*	[4]
199	126, *ADE1,2 cdc8-1*^{ts}	[4]
206	199, *SOE1*	[4]
126Δ12	126, *kti12Δ::KlLEU2*	This study
206Δ12	206, *kti12Δ::KlLEU2*	This study
206Δ12	206, *elp3Δ::KlLEU2*	[5]
RCY2866	*MAT***a** *ura3-52 leu2-3,112 SEC2*	[6]
RCY3256	RCY2866, *sec2-59* ^{ts}	[6]
RCY1903	RCY3256, *elp1Δ::URA3*	[6]
CMY85	RCY3256, *kti12Δ::URA3*	This study
ANY21	*MAT***a** *ura3-52 leu2-3, 112 trp1-289 his3 his4 suc gal2 SEC12*	[7]
MBY10-7A	ANY21, *sec12-4*^{ts}	[7]
CMY78	MBY10-7A, *elp1Δ::KlLEU2*	This study
CMY74	MBY10-7A, *kti12Δ::KlLEU2*	This study

Table S2. Primers.

Primer	Sequence (5′-3′)	Application
KTI12-P	tctcataccaaccggaaagg	seq *KTI12*
KTI12-1	ttgtcatcgtcatcgcgatg	seq *KTI12*
KTI12-2	ttcttactcctcaggacaac	seq *KTI12*
KTI12-3	aagcccttactcaacggatc	seq *KTI12*
KTI12-4	tacccagttgagaagacgag	seq *KTI12*
ko *KTI12* fw	aaactaaacaggcaatttagtaagaagatgccactggtgctttttacgggcgac	ko *KTI12*
ko *KTI12* rv	atctcaattcaagtttttgttaagataatcagcgaaaagcggaccgatccagct	ko *KTI12*
S3-*KTI12*	aggatcggtccgcttttcgctgattatcttaacaaaaacttgaatcgtacgctgcaggtcgac	et *KTI12*
S2-*KTI12*	atttcgtcttgccatttaccttctgatattaatcac atgtatatcatcgatgaattcgagctcg	et *KTI12*
S3-*kti12-2*	ctcaggacataactgactacatcgacgatatttgtaaagtagtctttcgtacgctgcaggtcgac	et *kti12-2*
S2-*kti12-2*	cccttactcaacggatcattgtgtccactgtttttagccgatttggaagtatcgatgaattcgagctcg	et *kti12-2*
S3-*kti12-3*	ccgatatcaatgatgatggttgcttcttcgtagacttgcccattggtaaccgtacgctgcaggtcgac	et *kti12-3*
S2-*kti12-3*	ttgaagttaatgaattgccttttcaatctctgcaattgcgccaacgtaacatcgatgaattcgagctcg	et *kti12-3*
up-*KTI12*	aagataggatcggtccgcttttcgctgattatcttaacaaaaacttgaattccatggaaaagagaag	tt *KTI12*
down-*KTI12*	agcaaatttcgtcttgccatttaccttctgatattaatcacatgtatatctacgactcactataggg	tt *KTI12*
KTI12-Pr-FW	cgccagtgctgcctctcttggtacg	ds *KTI12*
KTI12-PL-RV3	caaatgtttagcaagcgttgtcttaccactacatggttgcccacaaatcaccaccagtg	ds *KTI12*
KTI12-PL-FW3	cactggtggtgatttgtgggcaaccatgtagttagtggtaagacaacgcttgctaaaca	ds *KTI12*
KTI12-CBD-RV2	tgtggtggacaaatttttcacctcgcaccatagctcatatctgtaacctttgatactattcaacg	ds *KTI12*s
KTI12-CBD-FW2	cgttgaatagtatcaaaggttacagatatgagcctatggtgcgaggtgaaaaatttgtccaca	ds *KTI12*
KTI12-RV+50bp	tttcgtcttgccatttaccttctg	ds *KTI12*

Abbreviations: seq: DNA sequencing; ko: knock-out; et: epitope (c-Myc/HA) tagging; tt: TAP tagging; ds: domain swaps (P-loop or CBD) between yeast Kti12 and plant ELO4/DRL1 (see Figure 5).

Table S3. Plasmids.

Plasmid	Description	Source/Reference
YCplac33	Yeast-*E. coli* shuttle vector (Amp^R, *ARS1-CEN4*, *URA3*)	[8]
YCplac111	Yeast-*E. coli* shuttle vector (Amp^R, *ARS1-CEN4*, *LEU2*)	[8]
YEplac195	Yeast-*E. coli* shuttle vector (Amp^R, 2μ ori, *URA3*)	[8]
pCR2.1-TOPO	PCR cloning vector (Amp^R, Kan^R, *E. coli*)	Invitrogen
pJHW27	*KTI12* in YEplac195	[1]
pHMS14	Conditional expression vector (*GAL1*::γ-toxin, *HIS3*)	[9]
YDp-KlL/KlU	PCR template plasmids for gene deletion with Kl*LEU2*/Kl*URA3*	[9]
pYM1-5	PCR template plasmid series for C-terminal epitope tagging	[10]
pBS1479	PCR template plasmid for C-terminal TAP-tagging	[11]
pDJ40/16	*KTI12* in YCplac33/YCplac111	This study
pDJ75	*AtELO4/DRL1* in YCplac33	This study
pSS1/pSS9	*kti12-1* in pJJH27/YCplac33	This study
pSS1/pSS9	*kti12-1* in pJJH27/YCplac33	This study
pSS2/pSS10	*kti12-2* in pJJH27/YCplac33	This study
pSS3/pSS14	*kti12-3* in pJJH27/YCplac33	This study
pSS4/pUW72	*kti12-4* in pJJH27/YCplac33	This study
pSS5/pSS12	*kti12-5* in pJJH27/YCplac33	This study
pSS6/pSS13	*kti12-6* in pJJH27/YCplac33	This study
pSS7	*kti12-7* in pDJ16	This study
pSS8/puWK67	*kti12-8* in pDJ16/YCplac33	This study
pTU1	YCplac33 + *TDH3* promoter for constitutive gene expression	[12]
pTU1	*KTI12* in pTU1	G-T. Kim
pGTK101/111	*AtELO4/DRL1* in pTU1/YEplac195	G-T. Kim
pGTK102/112	*OsELO4DRL1* in pTU1/YEplac195	G-T. Kim
pGTK103/113	*PpELO4/DRL1* in pTU1/YEplac195	G-T. Kim
pHB17	*KTI12-c-Myc* in YCplac33	This study
pMW5	*KTI12-PLELO4-c-Myc* in YCplac33, (P-loop domain swap, Figure 5)	This study
pMW7	*KTI12-CBDELO4-c-Myc* in YCplac33 (CBD swap, Figure 5)	This study

2. Supplementary Figures

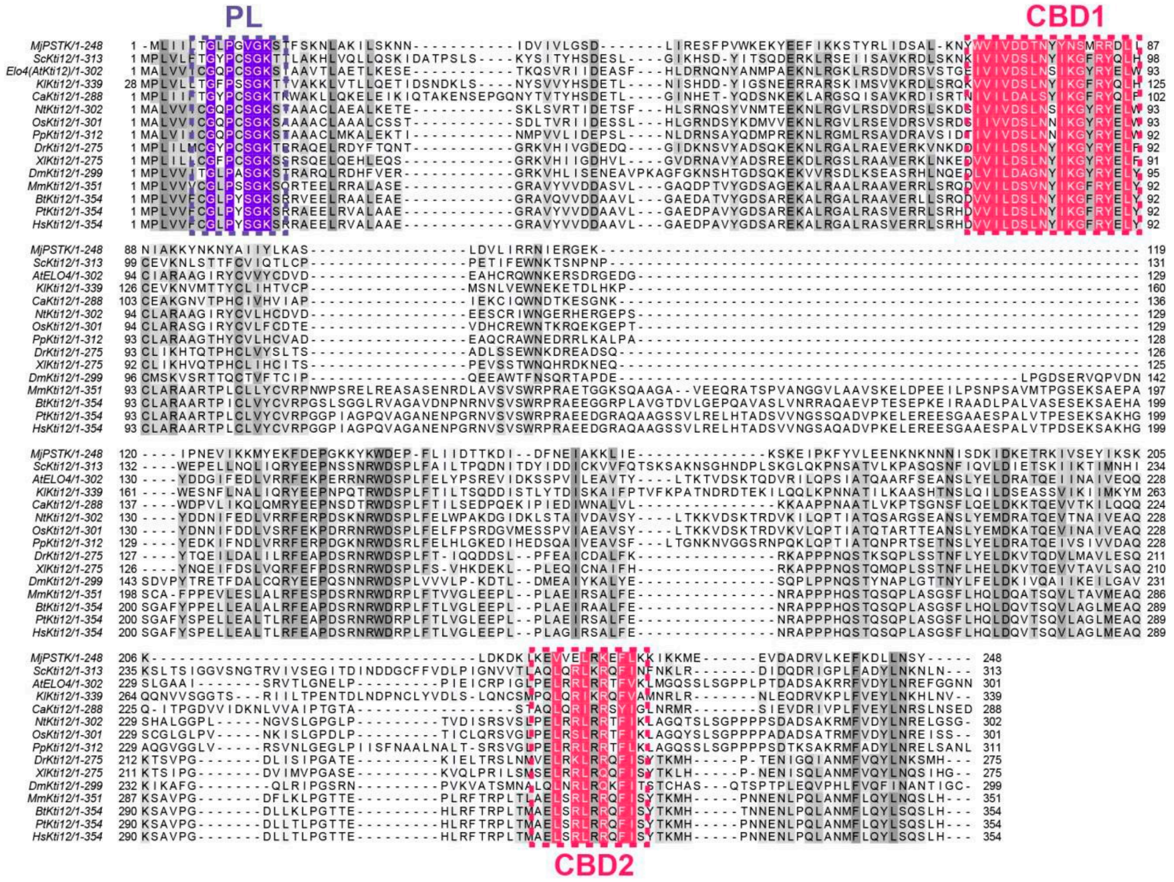

Figure S1. Multiple Kti12 sequence alignment. Main functional domains of Kti12 proteins are conserved in a variety of species and archaeal PSTK [*O*-phosphoseryl-tRNA(Sec) kinase]. Protein families (Pfam) database analyses and MUSCLE multiple alignment between archaeal PSTK (MjPSTK; Q58933) and Kti12 proteins from selected species reveals conserved protein motifs with putative functional roles in NTP (P-loop, PL) and CaM binding (CBD1 and CBD2). *Saccharomyces cerevisiae S288C* (ScKti12; NP_012812.1), *Arabidopsis thaliana* (ELO4(AtKti12); NP_172840.1), *Kluyveromyces lactis NRRL Y-1140* (KlKti12; XP_455212.1), *Candida albicans SC5314* (CaKti12; AOW31075.1), *Nicotiana tomensiformis* (NtKti12; XP_009612555.1), *Oryza Sativa* (OsKti12; XP_015615301.1), *Physcomitrella patens* (PpKti12; EDQ67537.1), *Danio rerio* (DrKti12; NP_001119890.1), *Xenopus laevis* (XlKti12; NP_001090073.1), *Drosophila melanogaster* (DmKti12; AAF45700.1 CG3587), *Mus musculus* (MmKti12; NP_083847.1), *Bos taurus* (BtKti12; NP_001074206.1), *Pan tryglodytes* (PtKti12; XP_009456012.1) and *Homo sapiens* (HsKti12; NP_612426.1).

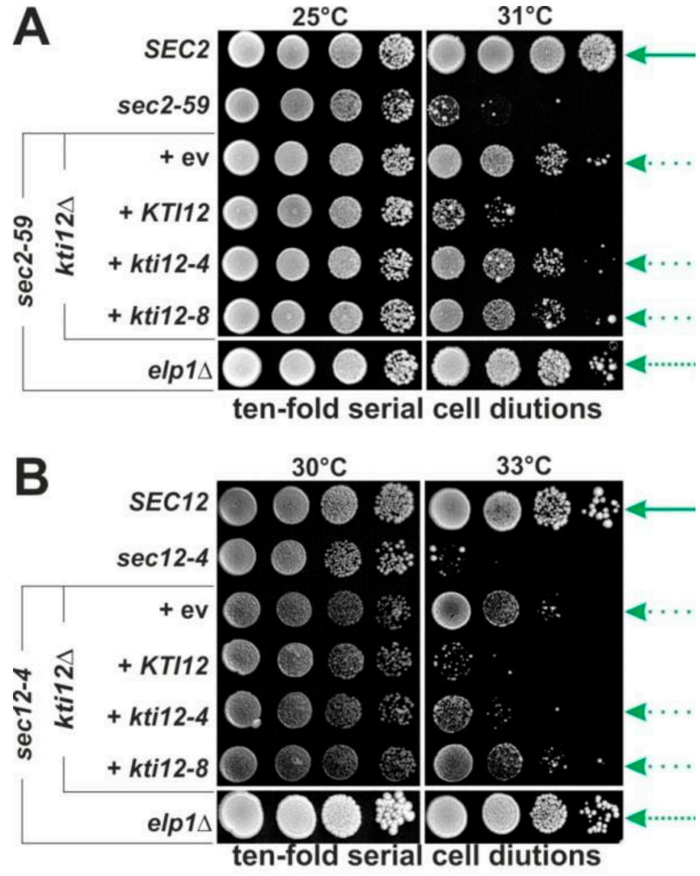

Figure S2. *ELP1* and *KTI12* mutations rescue thermosensitivity of *sec2-59*^{ts} and *sec12-4*^{ts} mutants. (**A**) Suppression of *sec2-59*^{ts}. Equivalent tenen-fold serial cell dilutions of the indicated strain backgrounds were cultivated at permissive (25 °C, left panel) and restrictive (31 °C) temperatures for 3 d. Growth rescue of *sec2-59*^{ts} by *ELP1* gene deletion (*elp1*) and partial suppression by *KTI12* gene mutations (*kti12*; *kti12-4*; *kti12-8*) is indicated (dotted arrows) in relation to wild-type *SEC2* growth (solid arrow); (**B**) Suppression of *sec12-4*^{ts}. Ten-fold serial cell dilutions of the indicated strain backgrounds were cultivated at permissive (30 °C, left panel) and restrictive (33 °C, left panel) temperatures for 3 d. Growth rescue of *sec12*^{ts} by *ELP1* gene deletion (*elp1*) and partial suppression by *KTI12* gene mutations (*kti12*; *kti12-4*; *kti12-8*) are indicated (dotted arrows) in relation to wild-type *SEC2* growth (solid arrow).

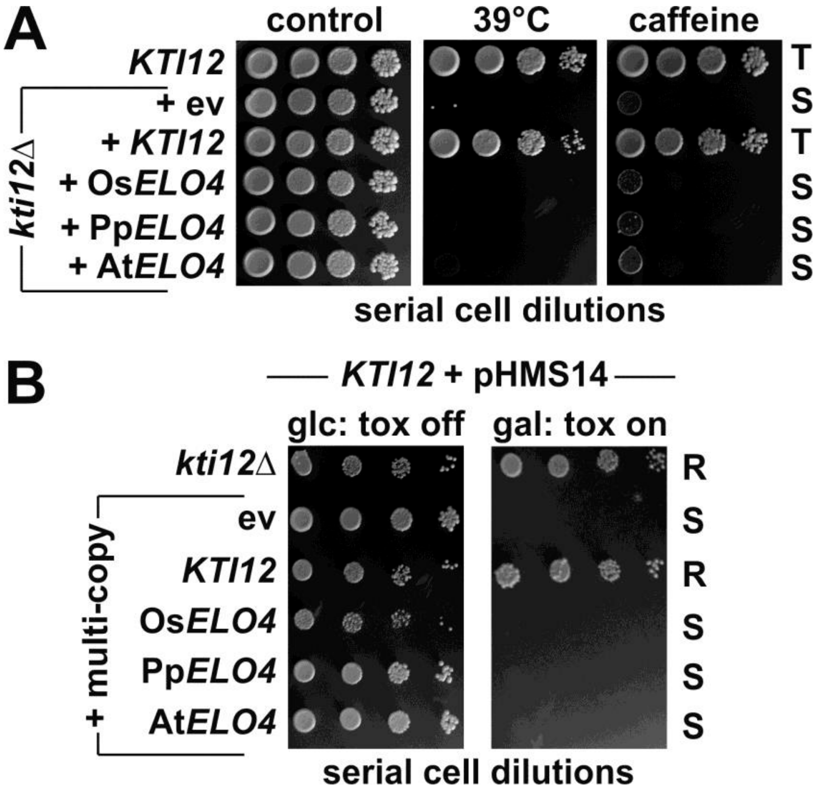

Figure S3. Analysis of yeast *kti12* cross-complementation by *ELO4* plant homologs. (**A**) Failure of single-copy *ELO4* from the three plant sources, i.e. from *Arabidopsis* (At*ELO4*), rice (Os*ELO4*) and moss (Pp*ELO4*), to rescue phenotypes triggered by a *kti12* knock-out mutation, i.e., inviability at 39 °C and sensitivity to caffeine. Equivalent ten-fold serial cell dilutions of the tester strains with the indicated genetic backgrounds were cultivated under standard (30 °C, left panel) or elevated temperatures (39 °C, middle panel) and in the presence of chemical stress (7.5 mM caffeine, right panel) and grown for 3 d. Inviability at 39 °C and sensitivity to growth inhibition by caffeine are denoted by 'S'; tolerance towards 39 °C and caffeine stress are indicated by 'T'; (**B**) Failure of multi-copy plant *ELO4* to induce resistance against expression of the -toxin tRNase from plasmid pHMS14 [6], which is typical of cells maintaining multi-copy *KTI12*. Equivalent ten-fold serial cell dilutions of the indicated tester strains were cultivated on glucose repressing (-toxin: off, left panel) or galactose inducing (-tox: on, right panel) media and grown for 3 d at 30 °C. Empty multi-copy vector control is abbreviated by 'ev'; Resistance/sensitivity towards conditional expression of zymocin's -toxin tRNase subunit on galactose is denoted by 'R/S'.

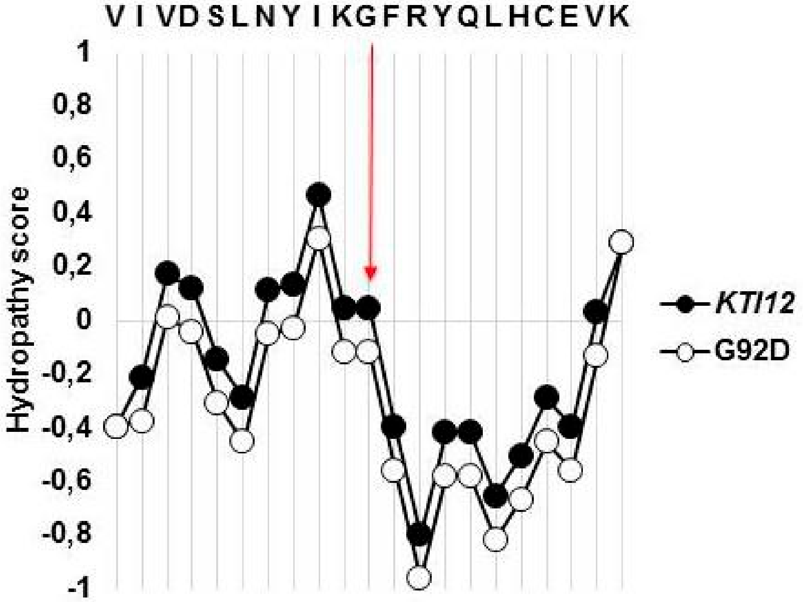

Figure S4. Hydropathy plots of Val-82 to Lys-102 spanning regions in Kti12 & Kti12-8. The plots were drawn using the method by Kyte and Doolittle [13] with ExPASy ProtScale (http://web.expasy.org/protscale/pscale/Hphob.Doolittle.html) and a window size of 19. The x axis indicates residues from Val-82 to Lys-102 in Kti12 with the Gly-92 residue (and its G92D substitution in Kti12-8) highlighted by a red arrow. The y axis indicates the relative hydrophobicity score of each residue in the context of full-length protein, where values above the midpoint line represent more hydrophobicity (internal sequences in the native protein) and ones below more hydrophilicity (external sequences in the native protein). Gly-92 physically localizes at the most hydrophilic surface in this region and the G92D substitution in Kti12-8 increases its hydrophobicity negative score suggesting the mutation renders the protein surface at this region more exposed.

References

1. Butler, A.R.; White, J.H.; Folawiyo, Y.; Edlin, A.; Gardiner, D.; Stark, M.J. Two *Saccharomyces cerevisiae* genes which control sensitivity to G1 arrest induced by *Kluyveromyces lactis* toxin. *Mol. Cell. Biol.* **1994**, *14*, 6306–6316.
2. Jablonowski, D.; Fichtner, L.; Stark, M.J.R.; Schaffrath, R. The yeast Elongator histone acetylase requires Sit4-dependent dephosphorylation for toxin-target capacity. *Mol. Biol. Cell* **2004**, *15*, 1459–1469.
3. Huang, B.; Johansson, M.J.; Bystrom, A.S. An early step in wobble uridine tRNA modification requires the Elongator complex. *RNA* **2005**, *11*, 424–436.
4. Su, J.Y.; Belmont, L.; Sclafani, R.A. Genetic and molecular analysis of the *SOE1* gene: A tRNA (3Glu) missense suppressor of yeast *cdc8* mutations. *Genetics* **1990**, *124*, 523–531.
5. Jablonowski, D.; Zink, S.; Mehlgarten, C.; Daum, G.; Schaffrath, R. $tRNA^{Glu}$ wobble uridine methylation by Trm9 identifies Elongator's key role for zymocin-induced cell death in yeast. *Mol. Microbiol.* **2006**, *59*, 677–688.
6. Rahl, P.B.; Chen, C.Z.; Collins, R.N. Elp1p, the yeast homolog of the FD disease syndrome protein, negatively regulates exocytosis independently of transcriptional elongation. *Cell* **2005**, *17*, 841–353.
7. Murakami, A.; Kimura, K.; Nakano, A. The inactive form of a yeast casein kinase I suppresses the secretory defect of the *sec12* mutant. *J. Biol. Chem.* **1999**, *274*, 3804–3810.
8. Gietz, R.D.; Sugino, A. New yeast-*Escherichia coli* shuttle vectors constructed with *in vitro* mutagenized yeast genes lacking six-base pair restriction sites. *Gene* **1988**, *74*, 527–534.
9. Frohloff, F.; Fichtner, L.; Jablonowski, D.; Breunig, K.D.; Schaffrath R. *Saccharomyces cerevisiae* Elongator mutations confer resistance to the *Kluyveromyces lactis* zymocin. *EMBO J.* **2001**, *20*, 1993–2003.

10. Knop, M.; Siegers, K.; Pereira, G.; Zachariae, W.; Winsor, B.; Nasmyth, K.; Schiebel, E. Epitope tagging of yeast genes using a PCR-based strategy: more tags and improved practical routines. *Yeast* **1999**, *15*, 963–972.
11. Puig, O.; Caspary, F.; Rigaut, G.; Rutz, B.; Bouveret, E.; Bragado-Nilsson, E.; Wilm, M.; Séraphin, B. The tandem affinity purification (TAP) method: A general procedure of protein complex purification. *Methods* **2001**, *24*, 218–229.
12. Jun, S.E.; Cho, K-H.; Hwang, J-Y.; Abdel-Fattah, W.; Hammermeister, A.; Schaffrath, R.; Bowman, J.L.; Kim, G.T. Comparative analysis of the conserved functions of *Arabidopsis* DRL1 and yeast KTI12. *Mol. Cells* **2015**, *38*, 243–250.
13. Kyte, J.; Doolittle, R.F. A simple method for displaying the hydropathic character of a protein. *J. Mol. Biol.* **1982**, *157*, 105–132.

© 2017 by the authors. Licensee MDPI, Basel, Switzerland. This article is an open access article distributed under the terms and conditions of the Creative Commons by Attribution (CC-BY) license (http://creativecommons.org/licenses/by/4.0/).
